# On intimate relationships between healthcare professionals and patients: a nationwide cohort analysis of medical tribunal decisions in the Netherlands

**DOI:** 10.1186/s12910-021-00628-0

**Published:** 2021-05-17

**Authors:** Wim Rietdijk, Sander Renes

**Affiliations:** 1 Department of Hospital Pharmacy, Erasmus University Medical Center, Doctor Molewaterplein 40, 3015GD Rotterdam, The Netherlands; 2 Department of Business Economics, Erasmus School of Economics, Erasmus University Rotterdam, Rotterdam, The Netherlands

**Keywords:** Medical disciplinary actions, Medical doctors & healthcare professionals, Professional behavior, Inappropriate sexual conduct

## Abstract

**Background:**

We examine the incidence of medical tribunal decisions and disciplinary actions (DAs) against healthcare professionals (HCPs). In addition, we studied whether an intimate relationship between an HCP and patient as part of the medical tribunal decision is associated with an increased likelihood of disciplinary actions.

**Methods:**

We conducted a nationwide cohort analysis on the downloadable medical tribunal decisions from a medical disciplinary tribunal in the Netherlands from 2010 to 2017.

**Results:**

We found that 117 (2.8%) of the 4,046 medical tribunal decisions involved an alleged intimate relationship between an HCP and patient. In these medical tribunal decisions the likelihood of a disciplinary action was significantly increased (odds ratio [OR] 12.97, 95% Confidence Interval [95% CI] 7.11–23.64). In addition, we found that nurses and psychiatrists are more frequently accused of and receive disciplinary actions due to intimate relationships than other HCP groups.

**Conclusions:**

We found a limited number of medical tribunal decisions involving an intimate relationship. Especially given the total number of medical tribunal decisions and the number of yearly HCP-patient interactions, the number appears small. Furthermore, an alleged intimate relationship or inappropriate sexual conduct is associated with an increased likelihood of disciplinary action. Future research should obtain statistics on the number of intimate relationships that actually start between HCPs and patients.

[Patient] indicated that he knew that he as a patient was not allowed to enter into a relationship with his healthcare professional, but that this prohibition applies to the defendant as a healthcare professional […] all the more because she should have realized that the [patient] was in a dependent situation.

*Freely translated from Dutch to English, ECLI:NL:TGZRZWO:2010:YG0026.*

## Background

In general, a treatment relation between patients and healthcare professionals (HCPs) starts with the patient presenting with a problem. Despite the inequality caused by the patient’s reliance on help from the HCP, treatment relations may evolve into intimate relationships or be marred by other sexual transgressions. The transgressions in treatment relations we found in this study ranged from sexual harassment to a one-night stand or a consensual long-term relationship. Furthermore, in a small number of medical tribunal decisions, HCPs are accused of serious transgressions, such as rape, child abuse, or the possession of child pornography. As the majority of medical tribunal decisions involving sexual transgressions involve sexual relationships between HCPs and patients, we will focus the discussion here on these types of relationships. The medical professional standards in the Netherlands prohibit any form of intimate relationship with a patient [1]. This prohibition is primarily because a patient-HCP treatment relation is typified by a power difference and a single-sided dependence. This dynamic makes it difficult for such a treatment relation to develop into an intimate relationship between equal partners.

Intimate relationships with patients are prohibited by professional standards for all HCPs in the Netherlands to protect both HCPs and patients [1]. The power asymmetry in the HCP-patient relation creates the need to protect patients against potential abuse fromHCPs. Similarly, the prohibition protects the professional image of HCPs, as well as the ability of HCPs to maintain an objective, professional stance with regard to the patient involved. The Dutch standards are very clear in this regard. Before any intimate relationship is started, the treatment relation has to be brought to an end, for instance, by referring the patient to another HCP. In addition, a cool-down period of several months is strongly advised for any post-treatment relation.

An intimate relationship can result in a conflict between the patient (or their representatives) and the HCP. An intimate relationship is sufficient grounds to bring the HCP before a medical disciplinary tribunal. A tribunal can impose disciplinary actions (DAs) ranging from formal warnings to prohibition to practice medicine. These conflicts can pose considerable professional risk, and a prohibition to practice will end the HCPs career [2, 3].

Previous studies have shown that DAs significantly impact the psychological and professional performance of HCPs [4–7] In addition, there is some debate as to which HCP specializations receive more complaints than others and what the reasons for the complaints are [8–10]. However, these studies were primarily performed in common law legal systems [8, 10–12] (e.g., the United Kingdom and USA) with a strong tradition of out-of-court settlements. The disciplinary system for HCPs in the Netherlands has similar goals to those of their international counterparts, such as medical licensing boards in common law countries. In the Netherlands disciplinary tribunals serve the dual functions of (1) specific prevention by correcting the behaviour of the healthcare professional involved—and (2) general prevention—by normatively describing and enforcing the professional standard [3, 7]. However, disciplinary tribunals are not used for restitution and reparation, as patients do not receive (financial) compensation through disciplinary tribunals [13]. Without strong financial incentives and within the civil law tradition of the Netherlands, settlements are rare. This makes it likely that we observe the relevant complaints when they arise.

To date, there has been no nationwide analysis of all medical tribunal decisions in civil law countries, such as the Netherlands. In particular, no evidence has been presented on the prevalence of medical tribunal decisions in general and specifically for intimate relationships with patients [6] or how these medical tribunal decisions are distributed over different HCP specializations. Therefore, the present study adds to the literature by examining the prevalence of allegations of intimate relationships in medical tribunal decisions. We will examine which HCP specializations have relatively more complaints and disciplinary actions in general and specifically for intimate relationships with their patients.

We are aware that medical tribunal decisions can represent significant and emotionally taxing events in the lives of the patients and HCP involved. Our aim is not to revive these events or conduct in-depth studies of individual medical tribunal decisions. We merely explored what the larger body of medical tribunal decisions can tell us about general patterns. By doing so, we hope to establish new insights into topics related to the professional behaviours of HCPs.

## Methods

### Data collection and the study population

We obtained data from the website of the Dutch medical disciplinary tribunal system through: https://tuchtrecht.overheid.nl.^14^ The data consisted of a description of the situation, evidence, and the decision made by the tribunal. Before publication of a decision on the website, all personal data of the patient and HCP were removed. This anonymization was conducted to protect the privacy of all parties involved. It was impossible to trace the medical tribunal decisions back to an individual patient and/or HCP, limiting the possibility of a more comprehensive analysis of medical tribunal decisions. As medical tribunal decisions are freely available, we were able to read the considerations of the tribunal for each medical tribunal decisions individually. As these decisions are freely available online no administrative permissions (e.g., informed consent to participate, consent from the disciplinary courts) were required to access the raw data. In contrast, we did request approval for the study by the local research board of our institution (ERIM Research review board; 2019/05/24-42345sre). However, it was impossible to download the body of medical tribunal decision as a single dataset. For this reason, we programmed a “web scraper”, i.e., an algorithm to download and structure the full texts of the medical tribunal decision from the website. This scraper was built in R studio (version 1.3.1093) using the ‘rvest’ package. We scraped medical tribunal decisions between January 1st, 2010 and December 31st, 2017.

**Fig. 1:**
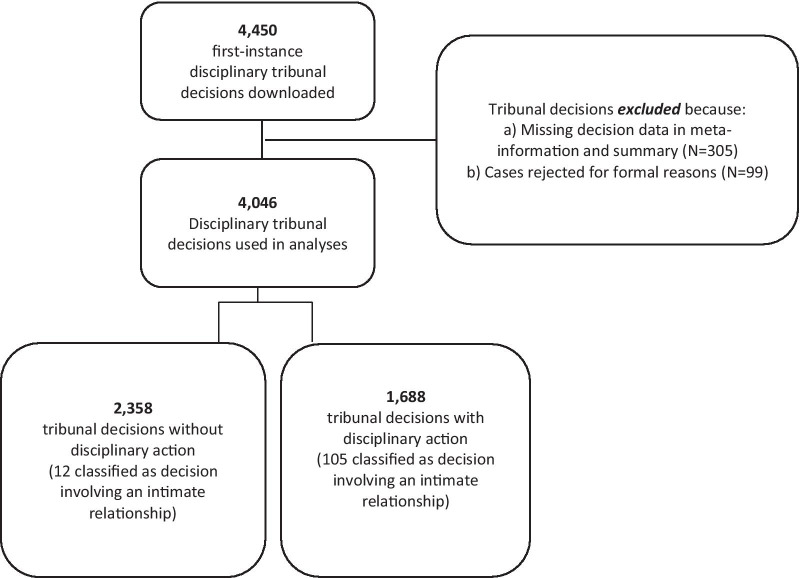
Flow chart

The website lists 4779 first-instance medical tribunal decisions of which we were able to scrape 4450 (93.1%). We did not study appeal decisions as these decisions only discuss points brought up in the first-instance and do not discuss alternative or additional complaints. The first-instance medical tribunal decisions therefore present a good overview of the number of complaints, reasons for starting a procedure and type of HCP involved. Figure 1 summarizes the steps taken to prepare the data for analysis purposes. Of the 4450 scraped medical tribunal cases, we were unable to find any information about the decision rendered by the tribunal in 305 (6.8%) cases, and these cases were removed before analysis. Furthermore, we excluded 99 medical tribunal decisions (2.2%) that were concluded on the basis of formal or legal procedural reasons, as these medical tribunal decisions do not revolve around patients, care, or HCP. After these steps, 4046 first-instance medical tribunal decisions were included in the statistical analysis. Of these 4,046 medical tribunal decisions, 1688 medical tribunal decisions involved at least one DA taken against the HCP, and 2358 medical tribunal decisions involved no DA.

As we used publicly available data that did not include confidential or personal information about the patient or HCP, medical ethical approval was not sought. However, the study was approved by the local Internal Review Board (2019/05/24-42345sre).

### Main outcome: disciplinary action (DA)

There were nine types of decisions present in our data: inadmissible (removed from analysis as they are concluded on formal reasons), rejected, admissible but no DA, warning and reprimand, temporary suspension, conditional practice, conditional suspension, prohibition to practice medicine, and prohibition to reregister. In our statistical analysis, we categorize medical tribunal decisions with outcomes ‘rejected’ and ‘admissible but no DA’ as no disciplinary action; the other medical tribunal decisions were categorized as a disciplinary action (DA).

### Independent variables: intimate relationship and type of healthcare professional

We used text analysis to identify medical tribunal decisions involving an intimate relationship or sexual misconduct (further referred to as intimate relationships). We use a wide definition of intimate relationships, ranging from sexual harassment, a one-night stand, to a consensual long-term relationship between HCP and patients. We searched for the terms ‘sex’, ‘sexual relationship’, and ‘intimate relationship’ in the main text of the medical tribunal decisions (in the Dutch language ‘*seks’*, ‘*sex’*, ‘*sexuele relatie’*, and ‘*intieme relatie’*).

Medical tribunal decisions that included these terms were subsequently screened independently by both authors (i.e., WR and SR). We screened for medical tribunal decisions where the intimate relationship was part of the complaints made against the HCP. Although minimal data were provided about the HCP or patient, we attempted to classify the medical tribunal decisions along three dimensions: (1) gender of the HCP, (2) whether the intimate relationship was consensual or nonconsensual, and (3) who initiated the medical tribunal case. The initiator of the medical tribunal case could be the patient, the Dutch healthcare inspection of other HCPs, a professional medical organization (e.g., the employer of the HCP), or an interested third part (e.g., the family of the patient). After the initial independent screening by both authors, we discussed each discrepancy to reach a consensus.

Second, we employed the classification of HCPs used by Statistics Netherlands (CBS, Centraal Bureau voor de Statistiek, Statline database) [15] to match medical tribunal decisions to specializations of HCPs. We downloaded the data on the number of people working in each specialization in the period 2010–2017 from the CBS website. For employment contracts the average full-time-equivalent (FTE) per specialization was calculated, whereas we assumed that all self-employed HCP worked full-time. We then matched the healthcare specializations in the medical tribunal decisions to the specializations for which the CBS keeps labour statistics.

### Statistical analysis

After scraping and cleaning the data, we analysed the data in three ways. As a first step, we calculated the incidence of medical tribunal decisions, disciplinary actions and medical tribunal decisions with intimate relationships per 1000 FTE years per specialization.

Furthermore, we summarized the medical tribunal decision involving intimate relationships after screening in three dimensions, gender of the HCP, initiator of the complaint, and proxy for the consensual nature of the intimate relationship, using numbers (and percentages, %).

Finally, we estimated a binary logistic regression using the presence of a DA as the dependent variable and the presence of an alleged intimate relationship in medical tribunal decisions as the independent variable. Additionally, we provided basic trends of the number of cases and DAs over the study period. We estimated the crude odds ratio (OR) and 95% confidence intervals (95% CI) using a p-value < 0.05 as statistically significant.

## Results

### Incidence of intimate relationships in medical tribunal decisions

Table 1 presents the results of the analyses of the distribution of disciplinary actions and intimate relationships with patients over the HCP specializations. We found that nurses and psychiatrists in particular had a relatively high incidence of medical tribunal decisions involving intimate relationships, with 3.42 and 0.99 cases per 1000 FTE working years, respectively.

**Table 1:** Incidence of medical tribunal decision and action also including numbers about intimate relationships per healthcare professional (HCP) specializations

	Total number of medical tribunal decisions			Per 1000 full time equivalent years		
Specialization	Medical tribunal decisions	DA	Intimate relationship	Medical tribunal decisions	DA	Intimate relationship
*Medical doctors (MD)*						
Psychiatry	391	147	12	32.26	12.13	0.99
Gynaecology	120	41	4	17.37	5.93	0.58
Plastic Surgery	38	14	1	20.42	7.52	0.54
Rheumatology	8	2	1	4.21	1.05	0.53
Urology	43	13	1	14.89	4.50	0.35
Gastroenterology	28	4	1	9.29	1.33	0.33
Dermatology	17	6	1	4.84	1.71	0.28
Surgery	320	108	2	30.37	10.25	0.19
General practitioner	831	329	12	9.84	3.90	0.14
Society & Health MD	378	150	1	27.51	10.92	0.07
MD Basic training	55	21	1	0.41	0.16	0.01
Pediatrics	63	17	0	6.59	1.78	0.00
Pathology	8	3	0	2.93	1.10	0.00
Internal medicine	135	30	0	9.35	2.08	0.00
Orthophedics	45	23	0	9.17	4.69	0.00
Cardiology	81	33	0	11.73	4.78	0.00
Radiology	29	9	0	3.79	1.18	0.00
Nucleair	2	2	0	1.72	1.72	0.00
Rehabilitation	16	5	0	4.55	1.42	0.00
Ophthalmology	46	14	0	10.38	3.16	0.00
Neuroloy	92	31	0	14.69	4.95	0.00
Microbiology	3	3	0	1.61	1.61	0.00
Radiotherapy	8	2	0	4.01	1.00	0.00
Otolaryngology	24	5	0	6.99	1.46	0.00
Clinical genetics	1	0	0	1.12	0.00	0.00
Anesthesiology	50	23	0	4.55	2.09	0.00
Pulmonary	30	12	0	7.29	2.92	0.00
MD specilaization handicaped patients	4	1	0	3.05	0.76	0.00
Chemist	0	0	0	0.00	0.00	0.00
Geriatrics	66	35	0	6.68	3.54	0.00
Other	93	40	5	10.50	4.51	0.56
Total medical doctors	2932	1083	42	7.71	2.85	0.11

	Total number of cases			Per 1000 full time equivalent years		
	Medical tribunal decisions	DA	Intimate	Medical tribunal decisions	DA	Intimate
*Other HCP*						
Nurse	292	135	35	28.56	13.20	3.42
Psychotherapist	66	52	9	2.07	1.63	0.28
Psychologist	193	111	9	2.56	1.47	0.12
Midwife	54	34	0	1.14	0.72	0.00
Dentist	311	183	6	4.71	2.77	0.09
Pharmacist	48	21	0	3.13	1.37	0.00
Physiotherapist	57	29	16	0.31	0.16	0.09
Total healthcare professionals	4046	1688	117	0.02	0.01	0.06

The first three columns list the total number of medical tribunal decisions, number of DAs and medical tribunal decisions involving intimate relationships per healthcare specialization as defined by Statistics Netherlands (CBS). The second three columns show the estimates of the number cases, DAs, and tribunal decisions involving intimate relationships per 1000 healthcare professional FTE years

### Describing medical tribunal decisions involving an intimate relationship

Table 2 presents a summary of the medical tribunal decisions involving an intimate relationship. Of the 4046 included medical tribunal decisions, we found 288 (7.1%) medical tribunal decisions to include our search terms. After screening these medical tribunal decisions, we found that 117 (2.8%) medical tribunal decisions involved an intimate relationship between HCPs and patients. Of these 117 medical tribunal decisions, 105 (89.7%) resulted in a disciplinary action against a HCP. Among the 117 medical tribunal decisions, 102 (87.2%) of the HCPs were male, 71 (60.7%) of the medical tribunal decisions involved a consensual relationship, and the vast majority of the medical tribunal decisions were initiated by either the patient (41, 35.0%) or the healthcare inspection (49, 41.8%). In Table 3 we present the descriptive statistics of medical tribunal decisions and DAs over the study period.

**Table 2:** Descriptive statistics of the medical tribunal decisions involving an intimate relationship (N = 117)

	N = 117
*HCP gender*	
Male	102 (87.2%)
Female	15 (12.8%)
Initiator of medical tribunal decision (claimant)	
Patient	41 (35.0%)
Healthcare inspection	49 (41.8%)
Medical professional organization	19 (16.2%)
Interested third party	8 (6.8%)
*Consensual relationship*	
Yes	71 (60.7%)
No	46 (39.3%)

Values are number (%) from the sample of screened medical tribunal decision

**Table 3:** Overview of medical tribunal decisions and disciplinary actions per year

	2010	2011	2012	2013	2014	2015	2016	2017
Intimate relationship	8	8	13	15	21	18	9	25
No intimate relationship	440	346	457	444	524	560	492	666
Fraction intimate relationship	1.82%	2.31%	2.84%	3.38%	4.01%	3.21%	1.83%	3.75%
Disciplinary action	194	149	182	195	220	265	201	282
No disciplinary action	254	205	288	264	325	313	300	409
Fraction disciplinary action	43.30%	42.09%	38.72%	42.48%	40.37%	45.85%	40.12%	40.81%
Total number of cases	448	354	470	459	545	578	501	691

### Intimate relationship and disciplinary action likelihood

The results of the binary logistic regression, see Table 4, indicate that there is a significant positive association between the presence of an intimate relationship and the likelihood of receiving a disciplinary action (OR 12.97, 95% CI 7.11–23.64) compared to medical tribunal decisions that do not include an intimate relationship.

**Table 4:** Binary logistic regression

	Outcome
	Disciplinary action (1 = yes)
Intimate relationship (1 = yes)	12.97 (7.11–23.64)
Total sample	4,046
Chi^2^ (df, p-value)	5375.0 (4044, p < 0.001)
AIC	5379.0

The estimate is an odds ratio (OR) and 95% confidence interval (95% CI)

## Discussion

The results from the present study showed that the incidence of medical tribunal decisions per 1000 FTE working years was approximately 0.2 for all HCPs. Furthermore, of the 4,046 medical tribunal decisions, 117 (2.8%) involved an intimate relationship between HCPs and patients, and 105 (89.5%) of these 117 resulted in a DA. In addition, we found that when a medical tribunal decision involved an alleged intimate relationship, the likelihood of a DA increased significantly.

Based on these results, one can argue that 117 medical tribunal decisions involving intimate relationships in the period 2010–2017 is a relatively small number. Particularly given the total number of medical tribunal decisions and the large number of HCP-patient interactions that occur on a daily basis. On the other hand, every intimate relationship is strictly prohibited in the Netherlands; thus, each intimate relationship is one too many. To increase the understanding of this topic, the actual number of intimate relationships between HCPs and patients in the Netherlands should be studied. Then, studying how many of these intimate relationships end in a tribunal decision may help to create a clear perspective. The comparison would make it possible to determine whether the patterns found in the disciplinary cases correspond to the patterns in all treatment relations. However, our data only show the number of cases in this last stage after a conflict is brought to a tribunal. Data on earlier steps of this transgression are unavailable.

The existing but older literature on sexual relations in the workplace generally focuses on the initial steps of sexual transgressions and on general office population [16]. This literature generally asks how many intimate relationships exist in the office. However, to the best of our knowledge, there are no proper estimates available for the later steps in the transgression with respect to HCPs, i.e., how many intimate relationships lead to a conflict and ultimately to a court or tribunal decision. In addition, general office populations are difficult to compare to HCPs, as intimate relationships are not formally regulated. These differences make it difficult to substantiate claims regarding whether the number of medical tribunal decisions and disciplinary actions is high or low.

We found that psychiatrists and nurses are most frequently involved in medical tribunal decisions involving an intimate relationship. However, the present data do not allow us to study the underlying reasons why these groups are found more frequently among medical tribunal decisions. For nurses, we may hypothesise that they are involved in the daily treatment of the patient and are “closer” to the patient than the doctor. Closer may refer to actual (reduced) physically distance but also to the mental support they provide patients. Similarly, for psychiatrists, we hypothesise that they typically help vulnerable patients with their mental health. Treatment requires psychiatrists to treat the psychological barriers of their patients and involves a more intensive interaction between HCPs and patients due to the nature of the treatment.

Finally, our results may contribute to the discussion of professional HCP behaviour in several ways. Discussion on more prevention interventions and schooling regularly occurs. However, given the low number of medical tribunal decision over a seven-year period, it is possible that more interventions to prevent intimate relationships may not be necessary. However, if one wants to establish an intervention, such an intervention may be targeted at specific specializations rather than at all specializations. Finally, the low number of medical tribunal decisions indicates that the transgressors are either very small in number, or very good at hiding their actions. In both cases, further prevention is likely better served by providing information to patients rather than targeting HCPs.

### Limitations

A potential limitation of the present study is the relatively small number of medical tribunal decisions involving intimate relationships that we found in the data, which prevented us from creating more comprehensive subcategories of intimate relationships. With so few observations, combining all types of transgressions together is the best available proxy for the classifications of medical tribunal decisions and disciplinary actions. Furthermore, our classification may have some limitations given that the search terms we used are limited. However, we used the most obvious terms referring to intimate relationships, and our manual screening of the identified decisions did not reveal any additional search terms.

### Future research

Future research might analyse these medical tribunal decisions in more depth. Our data showed that some HCP specializations have a relatively higher number of medical tribunal decisions than others. The question remains whether these specializations in turn also have a higher frequency of intimate relationships that do not result in a medical tribunal decision. If our data could be combined with actual numbers of intimate relationships between HCPs and patients (without a resulting conflict and medical tribunal decision), we could determine whether the patterns found in these medical tribunal decisions are an important signal of systemic problems on the aggregate level. Finally, future research may apply more advanced statistical techniques (for instance, using text-mining) to the data to uncover information from these medical tribunal decisions that we were not able to retrieve with the basic methods presented in this study.

## Conclusions

We found a limited number of medical tribunal decisions involving an intimate relationship. Especially given the total number of medical tribunal decisions and the number of yearly HCP-patient interactions, the number appears small. Furthermore, an alleged intimate relationship or inappropriate sexual conduct is associated with an increased likelihood of disciplinary actions. Future research should obtain statistics on how many intimate relationships actually start between HCPs and patients.

## Data Availability

The data of the present study are available from the corresponding author.

## Abbreviations

DA: Disciplinary action
HCP: Healthcare professionals
FTE: Full-time equivalent
CBS: Centraal Bureau voor de Statistiek (Statistics Netherlands)
OR: Odds ratio
95% CI: 95% confidence interval
